# Prevalence of long-term decision regret and associated risk factors in a large cohort of ICU surrogate decision makers

**DOI:** 10.1186/s13054-023-04332-w

**Published:** 2023-02-16

**Authors:** Sarah K. Andersen, Rachel A. Butler, Chung-Chou H. Chang, Robert Arnold, Derek C. Angus, Douglas B. White

**Affiliations:** 1 Program on Ethics and Decision Making, The Clinical Research, Investigation, and Systems Modeling of Acute Illness (CRISMA) Center, Department of Critical Care Medicine, University of Pittsburgh School of Medicine, 3550 Terrace St. Scaife Hall, Room 608, HPU010604, Pittsburgh, 15261 PA USA; 2 Palliative Research Center (PaRC), University of Pittsburgh, Pittsburgh, PA USA; 3 The CRISMA Center, Department of Critical Care Medicine, University of Pittsburgh School of Medicine, Pittsburgh, PA USA; 4 Division of General Internal Medicine, Section of Palliative Care and Medical Ethics, Department of Medicine, University of Pittsburgh School of Medicine, Pittsburgh, PA USA

**Keywords:** Intensive care, Surrogate decision-making, Patient-centered care, Psychological outcomes

## Abstract

**Background:**

Whether surrogate decision makers regret decisions about the use of life support for incapacitated, critically ill patients remain uncertain. We sought to determine the prevalence of decision regret among surrogates of adult ICU patients and identify factors that influence regret.

**Methods:**

We conducted a secondary analysis of data from the PARTNER 2 trial, which tested a family support intervention for surrogates of critically ill adults. At 6-month follow-up, surrogates rated their regret about life support decisions using the Decision Regret Scale (DRS), scored from 0 to 100, with higher scores indicating more regret. We used multiple linear regression to identify covariates associated with decision regret based on a psychological construct of regret. We constructed two models using the full cohort; model 1 included patient outcomes; model 2 focused on covariates known at the time of ICU decision-making. Subgroup analyses were also conducted based on patient survival status at hospital discharge and 6-month follow-up.

**Results:**

748 of 848 surrogates had complete DRS data. The median (IQR) DRS score was 15 (0, 25). Overall, 54% reported mild regret (DRS 5–25), 19% moderate-strong regret (DRS 30–100), and 27% no regret (DRS 0). Poor patient outcome at 6 months (death or severe functional dependence) was associated with more regret in model 1 (β 10.1; 95% C.I. 3.2, 17.0). In model 2, palliative care consultation (3.0; 0.1, 5.9), limitations in life support (LS) prior to death (6.3; 3.1, 9.4) and surrogate black race (6.3; 0.3, 12.3) were associated with more regret. Other modulators of regret in subgroup analyses included surrogate age and education level, surrogate-patient relationship, death in hospital (compared to the post-discharge period), and code status at time of ICU admission.

**Conclusions:**

One in five ICU surrogate decision makers experience moderate to strong regret about life support decisions in ICU. Poor patient outcomes are linked to more regret. Decisions to limit life support prior to patient death may also increase regret. Future studies are needed to understand how regret relates to decision quality and how to lessen lasting regret.

**Supplementary Information:**

The online version contains supplementary material available at 10.1186/s13054-023-04332-w.

## Introduction

Family and friends of critically ill patients are often called upon to act as surrogate decision makers for difficult decisions such as whether to withhold, withdraw, or intensify life support treatments. Surrogate decision makers are at high risk for long-term anxiety, depression, and post-traumatic stress disorder (PTSD) [1, 2], which may be driven in part by the act of making decisions [3, 4]. Decisions about life support treatments or end-of-life care appear particularly distressing and are associated with higher rates of PTSD and decisional conflict [3, 5]. Although the emotional and cognitive burdens of surrogate decision-making in the ICU are well-described in qualitative studies [6–9], large-scale data on validated measures of decision-making in this population are lacking. Better characterization of the decision-making process for surrogates may identify modifiable barriers to effective decision making, determine how decision-making contributes to psychological distress, and provide supporting evidence for provision of value-concordant care.

Decision regret is a psychological construct that is uniquely tied to decision making. Regret has been used for the past twenty years to evaluate high stakes health decisions in fields such as surgery and oncology [10–13]. Regret is defined as a negative emotion predicated on the belief that one’s present situation would have been improved by a different past decision, otherwise known as counter-factual thinking [10, 14]. Regret is often described as having two components: an affective experience that leads to emotional distress and a cognitive component that can inform future decisions [15].

Decision regret in non-ICU settings has been associated with discordance between preferred and actual decision-making role [11], perceived time pressures and incomplete information [12], lack of social support [10] and poor quality of life [16]. Decision regret has also been linked to poor psychological outcomes, particularly depression, although the direction of this association is unclear [10, 17]. Conversely, spirituality, values clarity, and shared decision-making have been associated with less decision regret [13, 18]. Large-scale studies are needed to better understand the prevalence of decision regret among ICU surrogates and the impacts of regret on surrogate well-being and delivery of goal-concordant care. Identification of risk factors for regret may also allow for targeted interventions to support high risk surrogates [19]. To this end, our study aims were to determine the prevalence of decision regret in a large cohort of surrogate decision makers of critically ill adult patients and identify factors associated with increased levels of decision regret.

## Methods

### Participant selection and data collection

To accomplish these aims, we conducted a cohort study using prospectively collected data from the PARTNER 2 trial (NCT02445937). PARTNER 2 (PAiring Re-engineered ICU Teams with Nurse-driven Education and Relationship-building) is a stepped wedge randomized control trial of a multicomponent nurse-led family support intervention for surrogate decision makers of adult ICU patients at high risk of death conducted at five ICUs in Pittsburgh, Pennsylvania [20]. The trial enrolled 848 patients and surrogates over four years between 2015 and 2019. Patients were enrolled if they were incapacitated and critically ill with a high risk of death or severe functional impairment (Additional file 1: Table S1). Eligible surrogates were those appointed in the patient’s advanced directives or, if no advanced directive existed, identified according to the hierarchy of surrogates codified in Pennsylvania state law. Surrogates were ineligible if under the age of 21, non-English speaking, or unable to complete study questionnaires due to mental or physical limitations. For this secondary analysis, surrogates were also excluded if responses to the decision regret questionnaire were incomplete.

As per trial protocol, data on exposures of interest for this study were collected directly from participants at enrollment (surrogate demographics), through chart abstraction following hospital discharge (patient demographics and illness information), and by phone interviews with surrogates at 6 months following the index hospitalization (patient outcomes). Data on surrogate decision regret, our outcome of interest, were also collected by phone at 6-month follow-up by assessors blinded to treatment group.

**Fig. 1:**
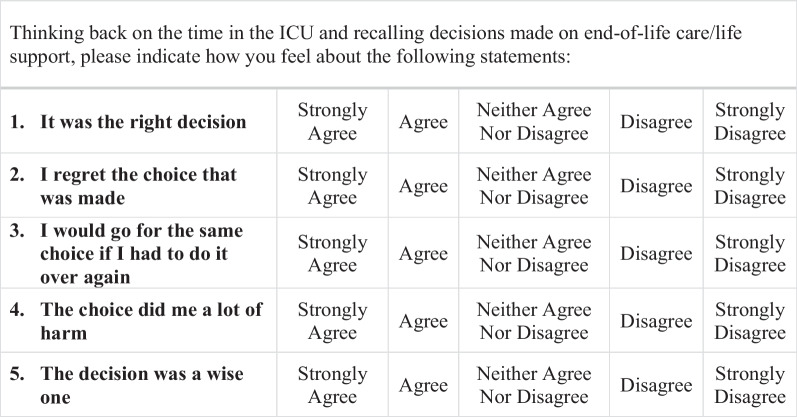
Decision Regret Scale (adapted for PARTNER 2) [9]

Surrogates were asked to score decision regret using the Decision Regret Scale (DRS), a validated 5-item measure of regret associated with past medical decisions [14]. Surrogates were specifically asked to consider decisions about life support or end-of-life care (Fig. 1). The DRS is one of three scales used for medical decision-making and was selected due to its applicability to past decisions and validation in multiple diverse patient cohorts [21, 22]. Items are scored using a Likert scale from 1 to 5 and then transformed into a final score ranging from 0 to 100 (increasing in intervals of 5), with higher scores indicating higher levels of regret. DRS scores are often divided categorically into no regret (0), mild regret (5–25), and moderate-strong regret (30–100) [23].

### Statistical methods

First, we determined the 6-month prevalence of decision regret among surrogates and conducted nonparametric testing to assess if median regret scores differed between intervention and control groups within the trial, as well as for surrogates whose loved one died in hospital or prior to 6-month follow-up. We then used multiple linear regression to identify explanatory factors that may influence decision regret. We decided that use of a multi-level model to adjust for clustering by trial site was unnecessary as intraclass correlation values were negligible when tested.

To create our multiple regression models, we identified variables of clinical interest a priori, drawing from a conceptual model of decision regret arising from the psychological literature on decision justification theory [19, 24, 25]. Decision justification theory proposes three forms of regret: process regret, option regret, and outcome regret. In other words, regret can arise from dissatisfaction with the decision-making process, dissatisfaction with the chosen option (regardless of outcome), or dissatisfaction due to an unfavorable outcome [25]. Using this conceptual model, we identified variables in our data set that related to the decision-making process (e.g., presence of advanced directives, prior surrogate experience, trial intervention), options chosen by surrogates (e.g., withdrawal of life support, change in code status), and outcomes (e.g., death, disability, patient location at 6 months). We also included patient and surrogate characteristics thought to be potential modulators of regret. The number of events per variable was greater than 15 for all models [26].

Missing data on 6-month location and functional status due to patient death, the largest cause of missingness, were handled using single-value imputation. Patients who died were assigned a 6-month location of ‘death’ and a Katz ADL score of 0, corresponding to ‘highly disabled’ (Additional file 1: Table S2) [27]. All missing data were assumed to be missing at random, and other sources of missingness, which were minor, were handled using complete cases analysis [28].

Two models were generated for the full cohort; the first included covariates related to patient outcome, while the second focused only on covariates known during the ICU admission. The purpose of this second model was to identify surrogates in the ICU setting who might be at especially high risk of regret and who may benefit from additional supports. Variance inflation factor testing was used to assess for collinearity and to refine our models [29, 30].

We further hypothesized that factors associated with regret may differ for surrogates whose loved ones died soon after critical illness. Thus, in addition to the full regression model, we also conducted stratified subgroup analyses according to patient survival at hospital discharge and 6-month follow-up. Given the smaller sample sizes for these models, backwards elimination at an alpha level of 0.5 was used to further refine variable selection. Sensitivity analysis was then performed by comparing variable selection models to complete models with all candidate variables included. All statistical tests were deemed significant at an alpha level of 0.05. Given the exploratory nature of our analyses, no correction for familywise error rate was performed [31, 32]. We performed all statistical analyses using Stata 16 (StataCorp, Texas, USA). This manuscript was written in accordance with the STROBE recommendations for reporting observational studies [33].

## Results

### Surrogate and patient characteristics

Out of 848 patient-surrogate dyads enrolled in the trial, 772 surrogates completed 6-month follow-up (Additional file 1: Fig. S1). Of those, 748 had complete decision regret scores and were included in the final analysis. Patient and surrogate characteristics are listed in Tables 1 and 2. Surrogates had a mean age of 56 years, 73% were female, 85% identified as White, and 72% were a child or spouse. Half had prior surrogate decision-making experience. Patients had a mean age of 64 years, 45% were female, and 80% identified as White. Just under half (40%) had completed an advanced directive prior to their index hospitalization, and 85% were full code at ICU admission. One third of patients died in ICU, and 92% of those had limitations of life support prior to death. At 6-month follow-up, 44% of the original cohort were still alive. Of those still alive, 77% were living at home, and 60% were functionally independent (Katz ADL score of 6).

**Table 1:** Surrogate characteristics

Surrogates	*N* = 748
*Age*	
Mean (SD)	55.9 (13.3)
*Sex*	
Male	202 (27.0)
Female	546 (73.0)
*Race*	
White	631 (84.5)
Black	91 (12.2)
Other	25 (3.3)
*Education*	
Did not graduate high school	41 (5.5)
High school diploma/GED	243 (32.5)
College or graduate school	460 (61.5)
Refused to answer	4 (0.5)
*Religious*	
Yes	631 (73.5)
No	222 (25.8)
Refused to answer	6 (0.8)
*Relationship to the patient*	
Spouse	291 (38.9)
Child	245 (32.8)
Parent	74 (9.9)
Other relative	131 (17.5)
POA/caregiver/friend	7 (1.0)
Past SDM^1^ experience	374 (50.0)

^1^*SDM* Surrogate decision-making

**Table 2:** Patient characteristics

Patients	*N* = 748
Age	
Mean (SD)	63.7 (15.0)
Sex	
Male	413 (55.2)
Female	335 (44.8)
Race	
White	598 (80.0)
Black	98 (13.1)
Other	52 (6.9)
APACHE II score at enrollment	
Mean (SD)	25.5 (7.4)
Full code at enrollment	636 (85.0)
Advanced directive	
Yes	443 (59.2)
No	298 (39.8)
No answer	7 (0.9)
Received mechanical ventilation	678 (90.6)
Underwent tracheotomy	188 (25.1)
Underwent PEG tube placement	110 (14.7)
Palliative care consultation	269 (36.0)
Died in hospital	267 (35.7)
Limitation of LST^1^ before death	*N* = 267
No	21 (7.9)
Yes	246 (92.1)
6-month mortality	
Alive	328 (43.9)
Dead	419 (56.1)
6-month location	*N* = 308
Home	236 (76.6)
Hospital	6 (2.0)
Skilled nursing facility	45 (14.6)
LTACH^2^	12 (3.9)
Inpatient rehabilitation facility	9 (2.9)
Katz Independence in ADLs^3^	*N* = 315
0	40 (12.7)
1	29 (9.2)
2	15 (4.8)
3	10 (3.2)
4	11 (3.5)
5	23 (7.3)
6	187 (59.4)

^1^*LST* Life-sustaining treatment

^2^*LTACH* Long-term acute care hospital

^3^ADL score of 6 = highly independent. Scored at 6-month follow-up

### Prevalence of decision regret

**Fig. 2:**
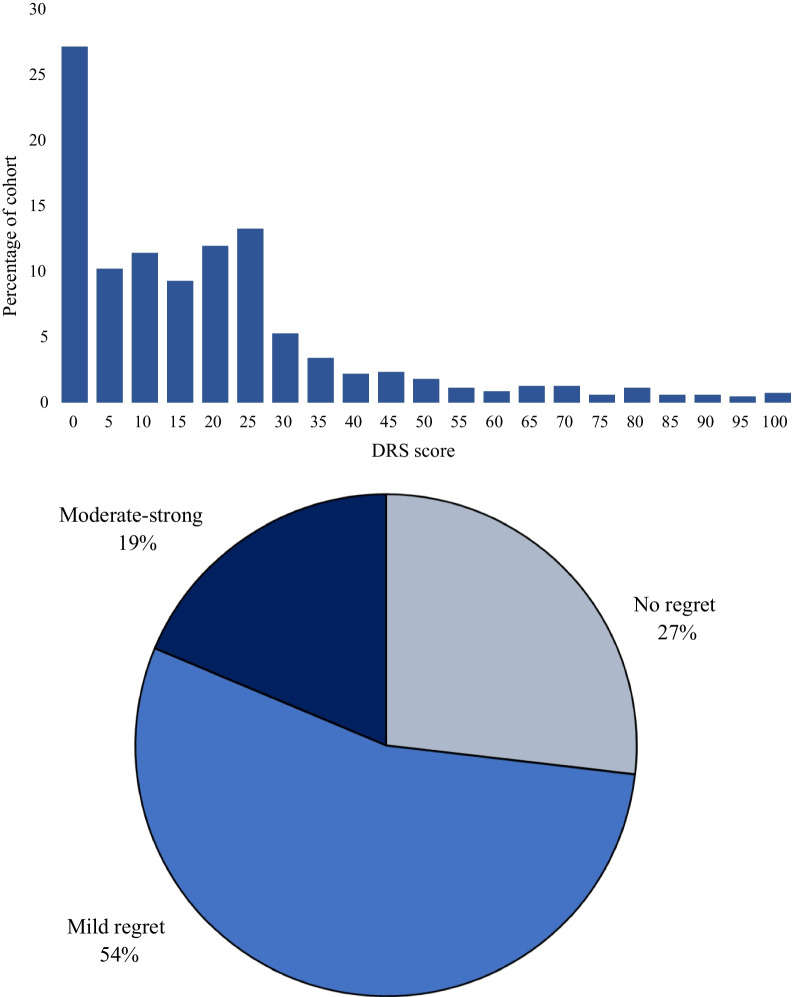
Prevalence of Decision Regret in ICU Surrogate Decision Makers

Decision regret scores for surrogates were positively skewed (Fig. 2) with a median (IQR) DRS score of 15 (0–25). Overall, 27% of surrogates reported no regret (DRS score 0), 54% reported mild regret (DRS score 5–25); and 19% reported moderate to strong regret (DRS score 30–100) (Fig. 2). Median DRS scores did not differ significantly for surrogates who received the PARTNER 2 intervention (compared to usual care) but were significantly higher for surrogates whose loved one died in the hospital (median [IQR] of 10 [0–25] for survivors versus 20 [5–25] for non-survivors; *p* < 0.0001) or prior to 6-month follow-up (10 [0–20] for survivors versus 20 [5–30] for non-survivors; *p* < 0.0001) when compared using the Wilcoxon rank-sum test﻿.

### Factors associated with regret in multivariable regression analysis

**Fig. 3:**
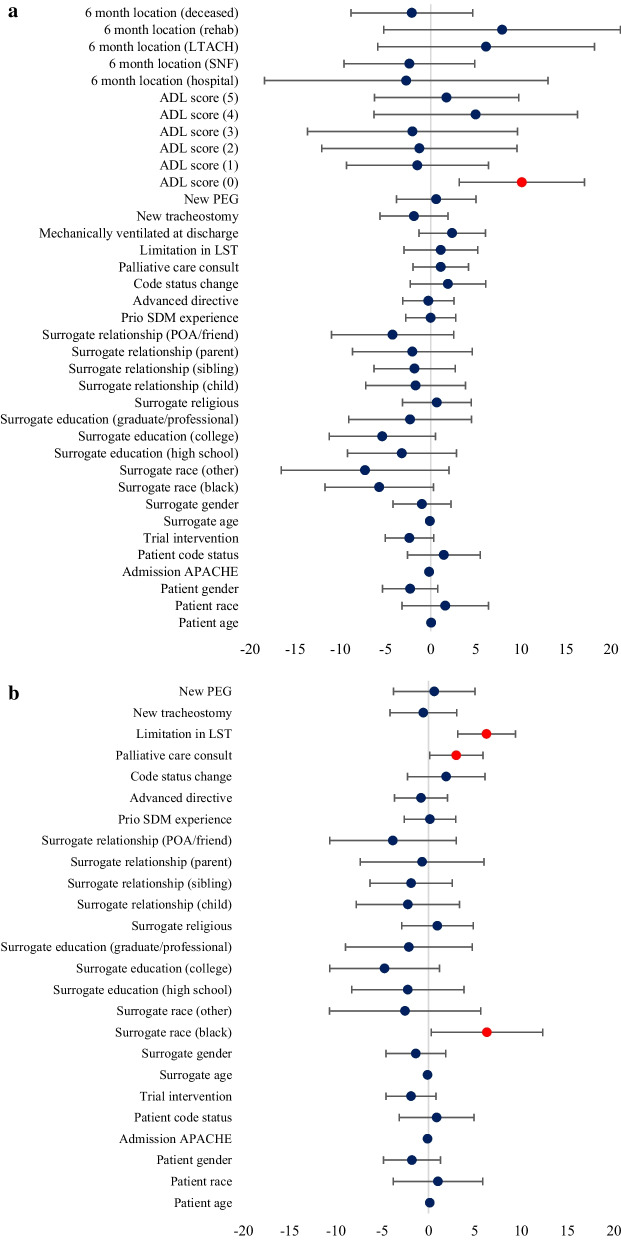
**a** Forest Plot of Multiple Regression Model 1 (Patient outcomes included). **b** Forest Plot of Multiple Regression Model 2 (Patient outcomes excluded)

Table 3 lists all covariates examined in our multiple regression analysis and more detailed definitions are provided in Additional file 1: Table S3. Covariates found to be significant in multiple regression analysis are shown in Table 4. For model 1 (patient outcomes included), only the composite patient outcome of death or severe disability at 6-month follow-up was significantly associated with regret (Fig. 3a). Surrogates of these patients had a 10.1-point increase in DRS scores (95% C.I. 3.2, 17.0). None of the other covariates had a significant effect on the likelihood of regret. Overall, model 1 had an adjusted R^2^ value of 0.06 and F-statistic of 2.17 (*p* < 0.001).

**Table 3:** Covariates included in multiple regression analysis

Category	Covariates	Data source
Demographics	*Patient* age, gender, race, APACHE II score, code status	Chart extraction
	*Surrogate* age, gender, race, education level, religion, relationship to patient	Enrollment questionnaire
*Decision justification theory (types of regret)*		
Process	Patient has advanced directive; surrogate had prior SDM^1^ experience	Enrollment questionnaire
	Palliative care consultation; PARTNER 2^2^ trial intervention	Chart extraction
Option	Limitation in life-sustaining treatments; change in code status; tracheostomy placement; PEG^3^ tube insertion	Chart extraction
Outcome	Patient discharged on mechanical ventilation; patient died in hospital; patient still alive at 6 months; patient functional status at 6 months (Katz ADL^4^ score)	6-month telephone interview

^1^*SDM* Surrogate decision-making, ^2^*PARTNER* PAiring re-engineered ICU teams with nurse-driven education and relationship-building, ^3^*PEG* Percutaneous gastrostomy, ^4^*ADL* Activities of daily living

**Table 4:** Factors that influenced regret in multiple regression analysis

Covariates	$\beta$ Coefficient^1^ (95% CI)	*p*-value
Full model with patient outcomes		
Death or functional dependence at 6 months^2^	10.1 (3.2, 17.0)	0.004
Full model with covariates available in ICU		
Surrogate black race	6.3 (0.3, 12.3)	0.04
Palliative care consultation	3.0 (0.1, 5.9)	0.04
Limitation in life sustaining treatments	6.3 (3.1, 9.4)	< 0.001
Stratified by patient survival to hospital discharge		
Survived		
Death after discharge or functional dependence	11.5 (6.0, 17.1)	< 0.001
Died		
Surrogate age (per year older)	− 0.3 (− 0.6, − 0.1)	0.02
Surrogate relationship to patient (vs. spouse)		
Child	− 8.7 (− 17.3, − 0.1)	0.05
POA^3^/caregiver/friend	− 13.2 (− 24.5, − 1.9)	0.02
Limitation in life sustaining treatments	10.1 (1.2, 19.0)	0.03
Stratified by patient survival at 6 months		
Survived:		
Full code at time of ICU admission	− 8.8 (− 16.8, − 0.85)	0.03
Functional dependence at 6 months	10.0 (2.0, 17.9)	0.01
Died:		
Surrogate age (per year older)	− 0.3 (− 0.5, − 0.1)	0.004
Surrogate education level (vs. less than high school)		
High school	− 6.0 (− 12.0, − 0.2)	0.04
College	− 6.9 (− 12.4, − 1.5)	0.01
Surrogate relationship to patient (vs. spouse)		
Child	− 7.0 (− 13.6, − 0.4)	0.04
POA/caregiver/friend	− 13.3 (− 22.1, − 4.5)	0.003
Limitation in life sustaining treatments	10.2 (1.0, 19.3)	0.03
Death in hospital (vs. after discharge)	− 10.1 (− 19.6, − 0.5)	0.04

^1^Increase in DRS score

^2^Measured using Katz Index of independence in activities of daily living

^3^*POA* Power of attorney for healthcare

For model 2 (patient outcomes excluded), three covariates were associated with more regret (β; 95% C.I.): palliative care consultation while in the ICU (3.0; 0.1, 5.9), limitations in life support prior to death (6.3; 3.1, 9.4), and surrogate black race (6.3; 0.3, 12.3) (Fig. 3b). Model 2 had an adjusted *R*^2^ value of 0.03 and F-statistic of 1.78 (*p* = 0.01).

When stratified by patient survival, surrogates with a loved one who was alive but severely disabled at 6 months had more regret (+ 10.0 points; 95% C.I. 2.0, 18.0) compared to those with less severe disability or who were functionally independent. Surrogates of survivors who were full code at the time of ICU admission had less regret than those of patients who had a DNR at time of ICU admission and survived (-8.8 points; 95% C.I. − 16.8, − 0.9).

For surrogates with a loved one who did not survive to 6-month follow-up, the following covariates were associated with less decision regret (β; 95% C.I.): patient death in the hospital (− 10.1; − 19.6, − 0.5) compared to death after hospital discharge; older surrogate age (− 0.31 points per year of age; − 0.5, − 0.1); surrogate graduation from high school (− 6.0; − 12.0, − 0.1) or college (− 6.9; − 12.4, − 1.5) compared to not finishing high-school, and being a child (− 7.0; − 13.6, − 0.4) or friend (− 13.3; − 22.1, − 4.5), rather than spouse of the patient. Conversely, limitation of life support treatments prior to death was associated with more regret than not limiting treatment (10.2; 1.0, 19.3).

Results were similar when stratified by death during index hospitalization (see Table 4). For patients who survived to hospital discharge, the composite outcome of death in the post-discharge period or severe disability at 6 months significantly increased surrogate regret (11.5; 6.0, 17.1). Except for code status in the 6-month survival group, subgroup results remained robust across sensitivity analyses.

## Discussion

Overall, we found that regret about life support decisions was common in a large cohort of surrogate decision makers for adult ICU patients. Almost three quarters of surrogates in our cohort experienced at least some regret, with one in five reporting moderate to strong regret at 6 months. Our results are similar to other studies with respect to median DRS scores, which ranged from 12.5 in surrogates of patients with intracerebral hemorrhage to 20 in caregivers of LVAD patients [5, 34, 35], and with respect to prevalence of regret [36, 37]. For instance, 72% of parents who decided to proceed with tracheostomy for a critically ill child had decision regret at 3 months [37] and 69% of surrogates of adult patients experienced regret following a tracheostomy or feeding tube decision [36]. In our study, poor patient outcomes (death or severe functional impairment) appeared most robustly associated with increased regret across multiple models. When patient outcomes were excluded from analysis, limitations in life support prior to death were also consistently associated with increased regret.

Although our regression analyses were exploratory in nature, many of the explanatory variables in our models are congruent with decision justification theory (DJT) and suggest potential opportunities for future interventions targeting regret. For instance, our finding that poor patient outcomes heavily influenced surrogate regret is supported by psychological research demonstrating that negative outcomes are strong drivers of outcome-based regret [19, 25, 38, 39]. According to DJT, outcome regret requires comparison to an alternate reference outcome, which is perceived to be superior. This alternative may be the person’s pre-decision status quo, their expected outcome, or the outcomes of others faced with a similar choice [19]. Recent work by Feiler et al. suggests that people feel more regret when they have less concrete knowledge of the foregone alternative, allowing them to imagine an infinite series of happier possible outcomes [40]. Thus, counseling surrogates on realistic outcomes after critical illness may be one way to decrease regret. Support groups that allow surrogates to learn about the experiences and outcomes of other families may be another tool.

Faced with the death of a loved one, how surrogates perceive decisions to withdraw or withhold life support may be particularly important. Feeling solely responsible for a difficult decision, particularly when the outcome is poor, may lead to increased emotional distress and self-blame [7, 19, 41]. Surrogates of non-survivors in our study may have felt greater regret following limitations in life support because they felt more directly responsible for the death of their loved one. If this hypothesis is correct, improved messaging and increased emotional support during discussions about withdrawal of life support may be beneficial to decrease feelings of guilt and regret.

Moreover, the dynamic connection between action, inaction, and regret may be relevant when it comes to end-of-life decision-making. Early on, people seem to regret action more than inaction, but the opposite becomes true as time passes [19]. For instance, in the study of LVAD patient and surrogate dyads, levels of regret differed within pairs and increased over time [34]. It is conceivable that regret related to limiting life support depends on how much time has passed since the decision was made. Framing comfort-focused care as an active intervention rather than passive process may also influence how surrogates view the decision to limit life support [42]. Further research is needed to determine how shared decision-making can be refined to lessen surrogate regret after decisions to limit life support treatments.

Regardless of outcome, people feel more regret if a decision is seen as unjustified or resulting from a faulty process [19, 36, 37]. Both surrogates and patients have more regret if they feel uninformed, pressured, or otherwise dissatisfied with their role in decision-making [37, 43]. Several additional modulators of regret in our study may relate to the decision-making process. For instance, more educated surrogates may feel less overwhelmed by medical information or have a more organized approach to decision-making [11]. Since behaving in line with one’s character or self-perception has been demonstrated to decrease regret [44], surrogates of patients who are full code and survive the ICU may feel better able to justify their decisions as value-congruent than surrogates of patients who have a DNR and receive aggressive ICU-level care. Surrogate-patient relationship is another factor that requires further study. It may be that non-spousal surrogates, and particularly non-relatives, are better able to emotionally distance themselves from the decision-making process or outcome.

Our finding that race was associated with regret may reflect a breakdown in the shared decision-making process related to physician biases or suboptimal communication. In other studies, clinician race bias has been associated with poorer quality communication and avoidance of advanced care planning [45, 46]. Conversely, bereaved surrogates of African American patients who died from a serious illness experienced less decisional conflict if they perceived the quality of communication as high [42]. Further work is needed to better understand the effects of racial factors on surrogate decision-making and regret.

The association between palliative care consultation and increased regret may reflect unmeasured psychosocial risk factors that led certain surrogates to receive palliative care consultation. Alternatively, there may have been some feature of the palliative care consultation process that unintentionally increased surrogate distress [47].

### Limitations

Our study has several limitations. First, the use of existing trial data did not allow us to examine all covariates that may influence decision regret, only those collected as part of the trial. Assuming missing data to be missing at random and the use of single-item imputation to handle missingness may have biased regression parameters and underestimated standard errors. We also lacked detailed information about the decisions surrogates considered when completing the DRS. Importantly, other studies have found that decision regret increased over time [34, 37]. Thus, the point at which we measured regret may have influenced our results. Serial measures of regret would help us better understand the surrogate experience. Our cohort was also enriched for patients with a high risk of poor outcomes and these results may not be generalizable to ICU surrogate decision makers in all circumstances. Surrogates who agree to participate in a clinical trial may also differ systematically from surrogates who do not participate, leading to volunteer bias. Lastly, our regression models were exploratory in nature and spurious results are a possibility. Further studies are necessary to refine our understanding of factors responsible for decision regret in ICU surrogates.

Measuring regret remains challenging. As a psychometric construct, the Decision Regret Scale focuses primarily on the option and outcome components of regret, rather than the decision process [21]. This feature of the scale may have influenced our finding that patient outcomes were most strongly associated with regret. Determining the threshold DRS score for clinically meaningful regret is also a challenge [23, 48]. A cut-off of 25 has previously been used to distinguish higher levels of regret without clear justification [23]. In a systematic review of medical decision-making studies, mean DRS score across studies was 16.5/100 and 4–20% of patients had scores over 25 [23]. Without an established gold standard measure, it is unclear if further scale calibration is needed or, less likely, if medical decision-making tends not to cause much regret. Additionally, the original DRS was not validated for use in surrogates [14]. A Decision Regret Scale for Caregivers (DRS-C) has subsequently been developed [48].

Finally, optimal measures of surrogate decision-making remain unclear [49]. Although decision regret causes psychological distress, it does not automatically follow that less surrogate regret indicates better decisions, particularly when considered from the patient’s point of view. Other measures of decision-making include decisional self-efficacy and decisional conflict [49]. Higher levels of decisional conflict have been associated with end-of-life decisions, whereas advanced directives have been associated with lower decisional conflict [5, 50, 51]. However, lower decisional conflict may also indicate a lack of engagement with hard choices, rather than better decision-making [52]. Moreover, decisional conflict is a transitional state with unclear long-term effects and an uncertain connection to decision regret [13, 53]. More work is needed to understand the links between decisional conflict, self-efficacy, and regret, as well as to determine what outcomes define success when it comes to surrogate decision-making in the ICU.

## Conclusion

We found that decision regret was common in a large cohort of surrogate decision makers for adult ICU patients. One in five had moderate to strong regret about life support or end-of-life decisions made 6 months prior. Poor patient outcomes are strongly associated with higher levels of regret, while limitations in life support may increase regret for surrogates of non-survivors. Future studies are needed to understand how regret relates to decision quality and how to lessen lasting regret.

## Supplementary Information

**Additional file 1:** PARTNER-2 Trial Data and Definitions.

## Data Availability

The datasets used and/or analyzed during the current study are available from the corresponding author on reasonable request.

## Abbreviations

ADL: Activities of daily living
C.I.: Confidence interval
DNR: Do not resuscitate
DJT: Decision justification theory
DRS: Decision regret scale
ICU: Intensive care unit
IQR: Interquartile range
LVAD: Left ventricular assist device
PTSD: Post-traumatic stress disorder
STROBE: Strengthening the reporting of observational studies in epidemiology
