# Supplementary material for: Prevalence of long-term decision regret and associated risk factors in a large cohort of ICU surrogate decision makers
Source: Crit Care. 2023 Feb 16;27:61. doi: 10.1186/s13054-023-04332-w (PMC9933411; doi:10.1186/s13054-023-04332-w)
Supplement: Supplementary file 1 — Additional file 1: PARTNER-2 Trial Data and Definitions. [file 13054_2023_4332_MOESM1_ESM.docx]

Additional File 1: PARTNER-2 Trial Data and Definitions

Supplemental Table 1: PARTNER-2 Eligibility Criteria

| **Patient Inclusion Criteria:**   - At least 21 years of age - Lack of decision-making capacity as determined by clinical examination by the attending physician - Presence of a medical illness associated with a high risk (at least 40% chance) of death or severe functional impairment   **Patient Exclusion Criteria:**   - No surrogate decision maker - Impending organ transplantation |
| --- |
| **Surrogate Inclusion Criteria:**   - Primary surrogate as determined by the patient’s advance directive or, if no directive exists, by following the hierarchy of surrogates codified in Pennsylvania state law - Up to 3 additional surrogates may be included per patient   **Surrogate Exclusion Criteria:**   - Under 21 years old - Unable to read and understand English - Unable to complete questionnaires due to physical or cognitive limitations |

Supplemental Figure 1: PARTNER-2 Consort Diagram

**Reason surrogate did not agree to approach by CRISMA Study Team (n=369)**

Not interested (n=**170**)

No surrogate available (n=**42**)

Previously enrolled (n=**25**)

Patient CTB before approached (n=**71**)

Patient d/c before approached (n=**56**)

Non-English Speaking (n=**3**)

PARTNER Champion did not approach (n=**4**)

Patients meeting eligibility criteria

(n=**1395**)

**Reason not consented (n =180)**

Refused (n=**108**)

Unable to reach for consent (n=**68**)

Surrogate Consented (n=**848**)

Surrogates of eligible patients who agree to approach by CRISMA Study Team (LTOC)

(n=**1024**)

Surrogate

Completed 6 month Follow-up

(n=**772**

Surrogate

Lost to

Follow-up

(n=**59**)

Surrogate

Refused Follow-up

(n=**15**)

Surrogate

Death prior to follow-up

(n=**3**)

Supplemental Table 2: Katz Index of Independence in Activities of Daily Living

| **Activity** | **Independence**  NO supervision, direction or personal assistance. | **Dependence**  WITH supervision, direction, personal assistance or total care. |
| --- | --- | --- |
| Bathing | (1 POINT) Bathes self completely or needs help in bathing only a single part of the body such as the back, genital area or disabled extremity. | (0 POINTS) Need help with  bathing more than one part of the body, getting in or out of the tub or shower. Requires total bathing |
| Dressing | (1 POINT) Get clothes from closets and drawers and puts on clothes and outer garments complete with fasteners. May have help tying shoes. | (0 POINTS) Needs help with  dressing self or needs to be  completely dressed. |
| Toileting | (1 POINT) Goes to toilet, gets on and off, arranges clothes, cleans genital area without help. | (0 POINTS) Needs help  transferring to the toilet, cleaning self or uses bedpan or commode. |
| Transferring | (1 POINT) Moves in and out of bed or chair unassisted. Mechanical transfer aids are acceptable | (0 POINTS) Needs help in moving from bed to chair or requires a complete transfer. |
| Continence | (1 POINT) Exercises complete self-control over urination and defecation. | (0 POINTS) Is partially or totally incontinent of bowel or bladder |
| Feeding | (1 POINT) Gets food from plate into mouth without help. Preparation of food may be done by another person. | (0 POINTS) Needs partial or total help with feeding or requires parenteral feeding. |
| **SCORING: 6 = High (patient independent) 0 = Low (patient very dependent** | | |

Supplemental Table 3: List of Covariates with Definitions

| Variable/ Field Name | Covariates | Field Attributes and Notes |
| --- | --- | --- |
| DRS100 | Decision regret | Decision Regret Scale (0-100); surrogate response at 6 months |
| surr_age | Surrogate age | continuous variable |
| surr_female | Surrogate gender | female(1), male(0) |
| race_cat | Surrogate race | Black(0), White(1), Other(2) |
| education_cat | Surrogate education | no HS(0), HS(1), college(2), graduate(3) |
| religion_binary | Does surrogate identify as religious? | yes(1), no(0) |
| relationship_cat | Surrogate relationship to patient | spouse(0), child(1), sibling(2), parent(3), other(4) |
| surr_mademeddecision | Does surrogate have prior SDM experience? | yes(1), no(0) |
| pt_age | Patient age | continuous variable |
| pt_white | Is patient white? | binary race variable; yes(1), no(0) |
| pt_advdirective | Does patient have an advanced directive | surrogate response on enrollment; yes(1), no(0) |
| resideprior | Where did patient live prior to admission? | home(1), nursing home(2), another person’s home(3), assisted living(5), group home(6), other(7) |
| died | Did patient die in hospital? | alive(0), died(1) |
| limitlifesustaintx | Was there any limitation of life-sustaining treatment prior to death? | yes(1), no(0)  ***only reported for patients who died in hospital*** |
| full_code | Was patient full code on trial enrollment? | yes(1), no(0) |
| codechange | Was there any change in code status during index admission? | yes(1), no(0) |
| new_peg | Did the patient receive a PEG during index admission? | yes(1), no(0) |
| trach | Did the patient receive a tracheostomy during index admission? | yes(1), no(0) |
| palliaorder | Was there a palliative care consultation? | yes(1), no(0) |
| stillalive | Was the patient alive at 6 month follow-up? | yes(1), no(0) |
| mechvent_hospdc | Was patient discharged on mechanical ventilation? | yes(1), no(0) |
| katz_ADLscore | KADZ index of activities of daily living | Functional outcome; categorical 0-6 (6=full independence)  ***only reported for patients alive at 6 months*** |
| location_6mo | Patient location at 6 month follow-up | home(0), hosp(1), SNF(2), LTAC(3),rehab(4)  ***only reported for patients alive at 6 months*** |
| intervention | Intervention or control arm | intervention(1), control(0) |
